# Influence of feedback characteristics on perceived learning value of feedback in clerkships: does culture matter?

**DOI:** 10.1186/s12909-017-0904-5

**Published:** 2017-04-05

**Authors:** Yoyo Suhoyo, Elisabeth A. Van Hell, Wouter Kerdijk, Ova Emilia, Johanna Schönrock-Adema, Jan B. M. Kuks, Janke Cohen-Schotanus

**Affiliations:** 1 Department of Medical Education, Faculty of Medicine, Universitas Gadjah Mada, Gd. Prof. Drs. Med. R. Radiopoetro, Lt. 6 Sayap Barat, Jl. Farmako, Sekip Utara, Yogyakarta, 55281 Indonesia; 2 Institute for Medical Education, University of Groningen and University Medical Center Groningen, Groningen, The Netherlands; 3 Department of Public an Individual Oral Health, Center for Dentistry and Oral Hygiene, University of Groningen and University Medical Center Groningen, Groningen, The Netherlands; 4 Education and Research Department, Hanze University of Applied Sciences, Groningen, The Netherlands; 5 Department of Obstetrics and Gynaecology, Faculty of Medicine, Universitas Gadjah Mada, Yogyakarta, Indonesia; 6 Department of Neurology, University Medical Center Groningen, Groningen, The Netherlands

**Keywords:** Feedback, Clerkship, Culture

## Abstract

**Background:**

Various feedback characteristics have been suggested to positively influence student learning. It is not clear how these feedback characteristics contribute to students’ perceived learning value of feedback in cultures classified low on the cultural dimension of individualism and high on power distance. This study was conducted to validate the influence of five feedback characteristics on students’ perceived learning value of feedback in an Indonesian clerkship context.

**Methods:**

We asked clerks in Neurology (*n* = 169) and Internal Medicine (*n* = 132) to assess on a 5-point Likert scale the learning value of the feedback they received. We asked them to record whether the feedback provider (1) informed the student what went well, (2) mentioned which aspects of performance needed improvement, (3) compared the student’s performance to a standard, (4) further explained or demonstrated the correct performance, and (5) prepared an action plan with the student to improve performance. Data were analyzed using multilevel regression.

**Results:**

A total of 250 students participated in this study, 131 from Internal Medicine (response rate 99%) and 119 from Neurology (response rate 70%). Of these participants, 225 respondents (44% males, 56% females) completed the form and reported 889 feedback moments. Students perceived feedback as more valuable when the feedback provider mentioned their weaknesses (β = 0.153, *p* < 0.01), compared their performance to a standard (β = 0.159, *p* < 0.01), explained or demonstrated the correct performance (β = 0.324, *p* < 0.001) and prepared an action plan with the student (β =0.496, *p* < 0.001). Appraisal of good performance did not influence the perceived learning value of feedback. No gender differences were found for perceived learning value.

**Conclusions:**

In Indonesia, we could validate four out of the five characteristics for effective feedback. We argue that our findings relate to culture, in particular to the levels of individualism and power distance. The recognized characteristics of what constitutes effective feedback should be validated across cultures.

## Background

To meet international quality standards for medical education, an increasing number of medical schools all over the world try to improve their curricula [1]. One of these standards addresses feedback in the clinical setting, because this kind of feedback is one of the most important tools to encourage student learning [2–6]. Medical schools are advised to ensure that students receive timely, specific, constructive and fair feedback to enhance their learning [1]. Several feedback characteristics have been described in literature as vital for the effectiveness of feedback [2–4, 6–15]. However, recent research indicates that the significance of feedback characteristics for the learning value of feedback may not hold for countries with different cultures [16]. We wondered whether feedback characteristics that were perceived to provide great learning value in a certain culture are also perceived to have great learning value in other cultures, and thus, whether feedback procedures that were developed in a certain culture can be implemented straight in a different culture. Therefore, in this study, we investigated how feedback characteristics, suggested in literature as essential for the effectiveness of feedback, influence Indonesian students’ perceived learning value of feedback.

Cultural differences seem to influence feedback processes and, therefore, may have implications for the applicability and effectiveness of feedback characteristics. In literature, it is suggested that the instructiveness of feedback is influenced by the status of the supervisor [3, 17], observation of behaviour [2, 18]. In the replication of a Dutch feedback study [14] in Indonesia [16], we considered the influence of the feedback provider and the role of direct observation on learning value, two recognized characteristics of effective feedback. Students were asked to register every feedback moment and asses the learning value of the feedback during a 2 - weeks period, and to report whether feedback was provided by a specialist who was perceived as an expert and as a credible person, and whether the feedback was based on direct observation. Overall, no differences in perceived learning value of feedback were found between the two countries. However, the two feedback characteristics under study were found to influence the perceived learning value differently, which could be explained from cultural differences between the two countries on the dimensions individualism and power distance [16, 19]. In Indonesia, a country classified as high on power distance [20], feedback provided by a specialist was perceived to have more learning value than feedback provided by a resident. In the Netherlands, classified as low on power distance [20], feedback from specialists and residents was perceived as equally valuable. In Indonesia, it did not make a difference whether the feedback was based on observation of the candidate or not. In the Netherlands, feedback based on observation was valued higher than feedback that had not been based on observation. It appears that the feedback requirements that should be met to optimize the effectiveness of the feedback differ between cultures.

The Indonesian replication study only took two feedback characteristics into account that were suggested as important for the effectiveness of feedback, namely the feedback provider and direct observation. However, five further feedback aspects have been described as essential for the effectiveness of feedback [2–4, 6–15]. First, students should be told what they have done well, to know their strengths [4, 7, 9, 12, 13]. Appraisal of good performance enhances students’ self-confidence and reinforces good practice. Second, students should be made aware of aspects of their performance that need to be improved [7, 9, 12, 13]. Information about performance deficiencies helps students to set learning goals. Third, students’ performance should be compared against a standard, such as a professional judgment, a local standard or existing guidelines [2, 4, 6, 8, 9]. This helps students to become aware of their progress towards mastery. Fourth, an explanation of the correct performance – what, how and why a task should be performed– should be provided to students to equip them with sufficient information to correct their errors [10, 15]. It is even more effective for student learning if the feedback provider models the correct performance [13]. Finally, the feedback provider should invite students to make a plan of action to improve their performance and discuss it with them [4, 6, 9, 12, 13]. Preparing plans for self-improvement helps students apply the feedback they received in practice and narrow the gap between actual and desired performance [4, 12].

Considering the differences between the two countries with regard to the importance of feedback provider and direct observation, the question arises what the influence is of the other five recognized feedback characteristics on Indonesian students’ perceived learning value of feedback, all the more since the importance of these aspects for the effectiveness of feedback has mainly been investigated and explored in countries classified as high on individualism (individualistic) and low on power distance. The aim of this study was to validate the importance of the other five characteristics for the effectiveness of feedback in a country low on individualism (collectivistic) and high on power distance. We investigated the influence of these characteristics on the perceived learning value of feedback in the context of the Mini Clinical Evaluation Exercise (mini-CEX). We formulated the following research questions:

1. How often do Indonesian students perceive the five internationally acknowledged characteristics of effective feedback during a mini-CEX?
2. How do these feedback characteristics influence Indonesian students’ perceived learning value of feedback?

## Methods

### Context

This study was conducted at the Faculty of Medicine, Universitas Gadjah Mada, Indonesia. The clinical phase consists of 2 years of clerkships in a department-based system. There are four eleven-week clerkship rotations in major clinical disciplines: Internal Medicine, Surgery, Obstetrics-Gynaecology and Paediatrics. There are seven four-week clerkship rotations in Neurology, Psychiatry, Dermatology, Ophthalmology, Otorhinolaryngology, Anaesthesiology, Radiology and Medical Forensics. Students do their rotations in two main teaching hospitals and several affiliated hospitals. Because we included only the departments where the mini-CEX was implemented, this study focused on the Internal Medicine and Neurology clerkships.

#### Mini-CEX

We decided to analyse the effectiveness of feedback characteristics in the context of the mini-CEX, which allows for and facilitates the application of all mentioned characteristics of effective feedback [21–26]. The mini-CEX focuses on observable competencies, is based on direct observation and provides a structure within which strengths, weaknesses and action plans can be discussed. The structure of the mini-CEX can facilitate the feedback provider in the role of expert and encourage comparison between students’ performance levels and performance standards. Consequently, the mini-CEX encourages the feedback provider to explain – on the basis of standards – what constitutes correct performance. Because of its potential, the mini-CEX has been used as a method to improve feedback during clerkships in many countries [27–29].

Students are obliged to have themselves assessed with the mini-CEX at least four times during the 11-week Internal Medicine clerkship, and at least twice during the four-week Neurology clerkship. The assessment form contains eight clinical competencies: history taking, physical examination, diagnosis, patient management, communication and counselling, professionalism, organization/efficiency and overall clinical care. Judgments are given on a 4-point scale (1 – under expectation, 2 – as expected, 3 – above expectation, and 4 – outstanding). Immediately after being observed, students are expected to ask a specialist for feedback and, subsequently, discuss their action plans to improve their performance. The two highest scores on the mini-CEX in each rotation are part of the summative, final grade at the end of the rotation. All assessment forms are recorded in the students’ logbooks, which they have to bring along during clerkships in all departments. Guidelines for all procedures, for both students and examiners, are also provided in the logbooks.

At all times, the mini-CEX examiners, to whom we will refer to as the feedback providers, are required to be specialists and clinical teachers at the main teaching hospital or one of the affiliated hospitals. All feedback providers had been introduced to the basic concepts of the mini-CEX (criteria and assessment procedure) and trained in providing constructive feedback.

### Participants and procedure

We asked students in Neurology (*n* = 169) and Internal Medicine (*n* = 132) to participate in the study. They were asked to assess the learning value of the mini-CEX feedback using a 5-point Likert scale ranging from 0 – not valuable to 4 – very valuable. Furthermore, we asked them to record for each mini-CEX whether the feedback provider:

1. informed the student what went well (‘strength’ variable);
2. mentioned which aspects of performance needed improvement (‘weakness’ variable);
3. compared the student’s performance to a standard, such as a protocol, guideline, standard of medical services, standard operating procedure or clinical skills’ book (‘comparison to standard’ variable);
4. explained the correct performance (‘correct performance’ variable);
5. prepared an action plan with the student to improve performance (‘action plan’ variable).

We have piloted the instrument and we have used these questions for our previous studies [14, 16]. Ethical approval for the study was obtained from the Medical and Health Research Ethics Committee (MHREC) at Universitas Gadjah Mada.

### Statistical analysis

Because each participant reported several and a varying number of feedback moments, the feedback moments needed to be dealt with as measurements that were nested within students. Because of this hierarchical data structure, we conducted a multilevel analysis. The analysis process comprised three stages. First, we established the necessity for multilevel analysis by calculating the Infraclass Correlation Coefficient (ICC). An ICC near zero would indicate no variance at the student level, while a higher number would indicate that variance in instructiveness stemmed from both the feedback moment level and the student level. Second, the empty model was estimated, which describes the variation in learning value associated with feedback moments and students separately. Third, the main effects model was used to calculate the impact of the feedback characteristics ‘strength’, ‘weakness’, ‘comparison to standard’, ‘correct performance’ and ‘action plan’ on the perceived learning value. The impact of the feedback characteristics was adjusted for gender and department, which were also added to the model. Subsequently, we examined the difference in deviance between models – the deviance assesses the extent to which a model deviates from the data – to compare the fit of the models with the data. Differences between models were determined using chi-square tests with the degrees of freedom being equal to the number of parameters added. We analyzed the data using MLwiN (version 2.01), a program specifically designed for multilevel analysis.

## Results

A total of 250 students participated in this study, 131 from Internal Medicine (response rate = 99%) and 119 from Neurology (response rate = 70%). Of these participants, 25 were excluded for having completed their forms inaccurately or incompletely. The remaining 225 respondents (44% males, 56% females) reported 889 feedback moments.

During the mini-CEX, the feedback providers mentioned students’ strengths in 59% and weaknesses in 72% of the feedback moments. Furthermore, they compared students’ performance to a standard in 49%, explained the correct performance in 68%, and prepared an action plan with the student to improve performance in 55% of the feedback moments.

The ICC was 0.38, indicating that 38% of variance in instructiveness was explained at the student level, which indicates that a multilevel approach was necessary. The overall mean of the perceived learning value of feedback was 3.5 on a 4-point scale (SE = 0.03). Students perceived feedback as more valuable when the feedback provider mentioned their weaknesses (*β* = 0.183, *p* < 0.01) and compared their performance to a standard (*β* = 0.174, *p* < 0.01). Explaining or demonstrating the correct performance (*β* = 0.322, *p* < 0.001) and preparing an action plan with the student to improve performance (*β* = 0.471, *p* < 0.001) both influenced the perceived learning value of feedback positively. Mentioning strengths did not influence the perceived learning value of feedback (*β* = −0.01, *p* > 0.05). We found no significant gender differences in students’ perceived learning value of feedback (*β* = 0.052, *p* > 0.05). Feedback received in the Neurology department was perceived to have a higher learning value than feedback received in the Internal medicine department (*β* = 0.151, *p* < 0.01). The main effects model fitted the data significantly better than the empty model (χ^2^(7) = 220; *p* < 0.001) (Table 1).

**Table 1:** Influence of different feedback characteristics and the receiver’s gender on the perceived learning value of the feedback expressed in regression coefficients and standard errors

Variables	Empty model	Main effects model
	Coeff. (SE)	Coeff. (SE)
Intercept	3.519** (0.043)	2.732** (0.086)
Strengths mentioned		−0.01 (0.06)
Weaknesses mentioned		0.183* (0.061)
Comparison of performance to standard		0.174* (0.056)
Explanation of correct performance		0.322** (0.053)
Action plan prepared		0.471** (0.055)
Department (0 = internal/1 = neurology)		0.151* (0.056)
Gender (0 = male/1 = female)		0.052 (0.082)
Variance		
Between students	0.244 (0.035)	0.245 (0.033)
Within students	0.387 (0.021)	0.291 (0.015)
Deviance	1928.225	1707.981**

*p < 0.01; **p < 0.001; *SE* = standard error

## Discussion

The aim of our study was to validate the importance of five feedback characteristics for the perceived the learning value of feedback in Indonesia, a country that is classified as low on individualism and high on power distance. All five feedback characteristics – ‘mentioning students’ strengths’, ‘mentioning students’ weaknesses’, ‘comparing students’ performance to a standard’, ‘explaining or demonstrating the correct performance’, and ‘preparing an action plan with the student’ – were perceived by the students, but not in all feedback moments. We found a positive influence of the latter four feedback characteristics, but mentioning strengths did not influence Indonesian students’ perceived learning value of feedback.

Teachers’ goals and expectations are important factors affecting student motivation for learning in cultures low on individualism and high on power distance [20, 30, 31]. Because of the power distance, students expect their teachers to determine the learning paths. This might explain the positive influence of four of the five feedback characteristics on Indonesian students’ perceived learning value of feedback. Students probably interpreted feedback based on these characteristics as outlines that serve to improve their learning and, thus, help them live up to their teachers’ expectations. The fifth characteristic of effective feedback – mentioning strengths – did not positively influence the perceived learning value of feedback, whereas, in literature, mentioning strengths has been perceived as conditional for feedback to be effective [4, 7, 9, 12, 13]. An explanation for this outcome may be that mentioning strengths does not relate to teachers’ goals and expectations. Students probably interpreted the reported strengths as no more than an act of kindness from the part of the feedback provider. Furthermore, in Indonesian society, there is a tendency towards modesty, which implies that not standing out from but fitting in with others is valued [31]. As such, compliments regarding personal performance may have little learning value for students.

We found that some feedback characteristics were more frequently used than others and we do believe that a cultural component is involved here as well. The characteristics ‘mentioning students’ weaknesses’ and ‘explaining or demonstrating the correct performance’ were used most frequently. Teachers can probably use these characteristics best to explain their expectations of the students. In countries classified as low on individualism and high on power distance, the goal of giving feedback is to correct errors and behaviour [32, 33]. Communicating clear expectations seems to be most effective to achieve this goal. The feedback characteristic ‘comparing performance to a standard’ was used least frequently in feedback. A possible explanation is that standards are perceived as just additional learning sources. Education in countries that are high on power distance, such as Indonesia, is teacher-centred [20, 30]. Teachers’ comments about students’ weaknesses and correct performance place them more in the centre of education than their comments about students’ performance in relation to the standards do. As such, teachers may feel that comparing performance to standards may be a less effective tool to communicate goals and expectations to the students. However, other factors may also influence the use of certain feedback characteristics. To get more insight into the feedback dynamics, we recommend qualitative research to explore why feedback providers attach more value to some than to other feedback characteristics and how this may be influenced by culture.

Seven feedback characteristics are described in literature as having a positive influence on student learning. In the current study, we examined five of these characteristics and in a former study, we investigated the other two [16]. Based on both studies, we conclude that – in the Indonesian context – two out of the seven characteristics did not positively influence Indonesian students’ perceived learning value of feedback: ‘direct observation’ and ‘mentioning strengths’. Besides, whether a feedback provider can serve as a good role model seems to be dependent upon the status of the feedback provider. Whereas in the Dutch context, it did not matter whether students received feedback from residents or specialists, in the Indonesian context feedback from specialists – higher in status than residents – was rated more positively than feedback from residents was. The differences between the findings described in literature and our outcomes can be explained by cultural differences, especially by differences with regard to the cultural dimensions individualism and power distance. More research on different cultures is needed to strengthen our outcomes.

A strength of our study is that we used the context of the mini-CEX, with trained specialists as feedback providers [29], to investigate the relations between feedback characteristics and students’ perceived learning value of feedback. The mini-CEX provides a structure that allows for and facilitates application of all mentioned characteristics of effective feedback [21–26]. Another strength is that the current study was performed in two departments – Internal Medicine and Neurology – in which the mini-CEX had been systematically implemented in the clerkship programme 2 years before [29]. We assumed that the specialists from these departments had become familiar with providing constructive feedback as they had been trained to do so and had already gained experience. However, further studies are needed to strengthen the generalizability of our results.

A limitation of our study is that only one Indonesian medical school was included. We are aware that our findings should be interpreted carefully because the cultural dimensions discussed could be a simplification of reality [34], since Indonesia is a very large country in which cultural conditions can differ from region to region [35]. However, in general, subcultures reflect the overarching national culture in which they are embedded, with cultural differences between countries being larger than those between subcultures within countries [34]. Therefore, our results may be generalizable to other medical schools in countries with a culture characterized by low individualism and high power distance. To strengthen the current outcomes, replication studies are needed.

Another limitation lies in the use of students’ reports and perceptions. We did not measure their actual learning. Even though perceived learning value is not the same as actual learning output, we feel perceptions of learning value provide a good indication of the quality of feedback, because positive perceptions by students are conducive to their learning processes [36]. As a corollary, positive perceptions of the learning value of feedback can be expected to improve learning and future performance. However, more research is needed to unravel the impact of feedback characteristics on students’ actual learning.

## Conclusion

In conclusion, some of the recognized feedback characteristics seem to be valid in different countries. However, the learning value of the characteristics ‘feedback provider’, ‘observation’ and ‘mentioning strengths’ seems to depend on the prevailing culture of countries, in particular on the levels of individualism and power distance. Therefore, we recommend that culture should be considered when implementing international standards for feedback in order to optimize student learning and development. We also suggest to further validate our findings in different cultural settings in order to improve our understanding of what constitutes effective feedback in different cultures and why.

## Abbreviations

Coeff: Coefficient
ICC: Intraclass Correlation Coefficient
Mini-CEX: Mini Clinical Evaluation Exercise
SE: Standard error
